# Nine-Month Continuous Fremanezumab Prophylaxis on the Response to Triptans and Also on the Incidence of Triggers, Hypersensitivity and Prodromal Symptoms of Patients with High-Frequency Episodic Migraine

**DOI:** 10.3390/jcm13020386

**Published:** 2024-01-10

**Authors:** Emmanouil V. Dermitzakis, Michail Vikelis, Georgia Xiromerisiou, Dimitrios Rallis, Panagiotis Soldatos, Pantelis Litsardopoulos, Dimitrios Rikos, Andreas A. Argyriou

**Affiliations:** 1 Euromedica General Clinic, 54645 Thessaloniki, Greece; 2 Headache Clinic, Mediterraneo Hospital, 16675 Athens, Greece; mvikelis@headaches.gr; 3 Department of Neurology, University Hospital of Larissa, University of Thessaly, 41110 Larissa, Greece; georgiaxiromerisiou@gmail.com; 4 Department of Neurology, Tzaneio General Hospital of Piraeus, 18536 Athens, Greece; jimrallis@hotmail.com; 5 Independent Researcher, 24100 Kalamata, Greece; soldatosp@gmail.com; 6 Headache Outpatient Clinic, Department of Neurology, Agios Andreas State General Hospital of Patras, 26335 Patras, Greece; pantelis84@hotmail.com (P.L.); andargyriou@yahoo.gr (A.A.A.); 7 404 Military Hospital, 41222 Larisa, Greece; rikosd@hotmail.com

**Keywords:** fremanezumab, effects, response to triptans, hypersensitivity symptoms, prodromal symptoms, triggers

## Abstract

**Objective:** To investigate whether the incidence of triggers, prodromal symptoms, hypersensitivity symptoms accompanying headache and responses to triptans were modified during a continuous 9-month fremanezumab therapy for migraine prophylaxis. **Patients and methods:** We studied 63 patients with high-frequency episodic migraine (HFEM). Enrolled patients received fremanezumab for nine consecutive months before defining the response rates and being stratified into treatment responders (≥50–74% reduction in monthly headache days (MHDs)), super responders (≥75%), partial non-responders (<50%) and super non-responders (<30%). Through headache diaries, patients provided data in order to document the impact of fremanezumab on the incidence of triggers, associated symptoms followed by headache and response to triptans (the use of the migraine treatment optimization questionnaire-4 (mTOQ-4)) during the 9-month treatment period. **Results:** Fremanezumab had early (after 3 monthly cycles) beneficial effects on the response to triptans in the majority of responders with relevant increases in mTOQ-4 scoring, but also in half of partial non-responders. A significant reduction in median days with migraine-associated symptoms was seen in responders after 6 months of therapy with fremanezumab, mostly for osmophobia, photophobia, phonophobia and nausea/vomiting, but partial non-responders also benefited. Likewise, the incidence of self-reported prodromal symptoms was significantly reduced in responders and was modestly diminished in partial non-responders. Triggers remained unaffected in both responders and non-responders. **Conclusions:** Fremanezumab given for at least 6–9 months may exert neuromodulatory effects in the migraine brain. These effects could result both in the inhibition of migraine chronification, but also in the diminishing of the magnitude of migraine-associated symptoms, mostly in responders and in partial non-responders.

## 1. Introduction

Based on the frequency of its attacks, migraine can be classified into episodic (<15 monthly headache days (MHDs)) or chronic migraine with an occurrence for more than three consecutive months of ≥15 MHDs, of which at least eight have typical migraine features. For further classification but also for practical purposes, the episodic form of migraine can be further subdivided into low-frequency (i.e., 4–7 days/month) and high-frequency episodic migraine (HFEM), where the MHDs are between 8 and 14 [1].

Attacks of head or facial pain are the core phenotypic element of migraine, but several other symptoms may exist during the timeline of a migraine attack as the phenomenon evolves over time. Towards the latter view, it is recognized that a migraine attack can be initiated through the prodromal and aura phase, evolve to its ictal state and resolve during the migraine postdrome [2]. Prodromal (premonitory) symptoms may precede the headache phase by up to 72 h, with manifestations of various symptoms, including mood changes, yawning, somnolence, drowsiness, food craving, neck stiffness and fatigue [3]. Some environmental stimuli, such as weather changes, dehydration, consumption of certain food/drinks, psychological distress or irregular sleeping patterns may be able to individually trigger a migraine attack and its prodromes [4]. During a migraine attack, apart from headache, several other hypersensitivity symptoms may frequently occur and have been self-rated by patients as being more disabling than the headache itself [5,6].

The recognition of the key role that the calcitonin gene-related peptide (CGRP) receptor plays in the pathophysiology of migraine, eventually leading to the development of CGRP inhibitors, has revolutionized the prophylactic treatment of migraine. CGRP is a 37-amino-acid neuropeptide with potent vasoactive properties [7]. It has been previously demonstrated that there is evidence of CGRP release and abundant vasodilation in the dura matter and pial arterial vasculature under conditions of neurogenic inflammation to eventually activate the trigeminovascular nociceptors and generate headache [8,9]. Based on the concept that blocking CGRP release from peripheral nerve terminals of meningeal and trigeminal nociceptors may prevent central sensitization [10], researchers have developed four anti-CGRP monoclonal antibodies (anti-CGRP Mabs). Among these anti-CGRP Mabs, erenumab targets the CGRP receptor, while fremanezumab, galcanezumab and eptinezumab target the CGRP ligand, thoroughly differing in their evoked brain responses [11]. 

It has been recently demonstrated that anti-CGRP Mabs are able to evoke changes in the hyperexcited migraine brain, thoroughly offering adequate migraine prophylaxis but also improvements in the premonitory and accompanying symptoms of migraine [12,13]. We have previously demonstrated that fremanezumab was able to significantly reduce MHDs and also had other efficacies such as in disability and quality of life outcomes in HFEM patients [14], in agreement with the results of another real-life study in a large sample of patients treated with fremanezumab that likewise demonstrated an early and sustained efficacy in HFEM patients with multiple preventive treatment failures [15]. However, a recent study has demonstrated that galcanezumab was able to quiet the abnormal hyperexcitability characteristic in the brains of patients with migraine, thoroughly offering MHD reductions but also improvements in migraine triggers and prodromal symptoms [13]. 

In order to seek if similar beneficial effects occur with fremanezumab, we studied HFEM patients and investigated whether the incidence of triggers, premonitory symptoms, hypersensitivity symptoms accompanying headache and response to triptans would change during a continuous 9-month therapy for migraine prophylaxis.

## 2. Materials and Methods

For the purposes of the current open-label prospective analysis, a total of 63 adult patients with a definite diagnosis of HFEM were included [1]. Enrolled patients were treated with monotherapy with 9 continuous monthly cycles of fremanezumab for their migraine prophylaxis. The Institutional Review Board of “Agios Andreas” Patras General Hospital granted approval to the study protocol, while patients provided their informed consent for participation before their study entry.

No statistical power calculation was conducted prior to the study and the sample size was based on a previous study with a similar design [13]. Eligibility was confirmed with a protocol-specific checklist, while the general inclusion and exclusion criteria were the same as those that have been previously described in detail [14,16,17]. Briefly, anti-CGRP-Mabs-naïve patients aged above 18 years had to suffer from HFEM with or without aura or medication-overuse headache (MOH) and be scheduled to receive monotherapy with fremanezumab for their migraine prophylaxis, in line with the current Greek reimbursement policies. According to the latter policies, currently applied for high-cost therapies, fremanezumab can only be approved in the context of a full reimbursement by the Greek National Health System in HFEM patients (submission of application from the treating physician and independent evaluation through the Electronic Prior Authorization System) who fail to respond to or did not tolerate at least three previous first-line oral preventatives. Patients were excluded if they were with the presence of a major psychiatric disorder, e.g., psychosis, pregnancy or any contraindication to fremanezumab, according to the approved summary of its characteristics [17]. 

Enrolled patients received subcutaneous fremanezumab (Ajovy^®^ 225 mg/pf-syr, Teva Pharma-Greece) 225 mg monthly (every 28–30 days) for nine consecutive months before defining the response rates and being stratified into groups according to the following grading: (i) responders (50–74% reduction in MHDs, compared to baseline); (ii) partial non-responders (30 to 49% reduction in MHDs); (iii) super responders (≥75% reduction in MHDs) and (iv) super non-responders (<30% reduction in MHDs) [18]. The response to treatment was established by estimating the reduction in MHDs at the clinical follow-up, which was performed 9 months after fremanezumab initiation, compared to baseline (28-day pre-treatment period), but the relevant efficacy data were also obtained at month 3 and 6. The response to treatment was established with the use of headache diaries.

At baseline and at each subsequent follow up at month 3, 6 and 9, patients were asked to provide data (all in categorical yes/no paper format) concerning the following clinical variables throughout the treatment period: (i) response to triptans, defined as headache resolution within 2 h after triptan intake. This outcome was quantified with the use of the migraine treatment optimization questionnaire-4 (mTOQ-4), which is a validated self-report questionnaire used to assess the optimization of acute treatment in persons with migraine ranging from 0 to 8 and higher scores indicating better acute medication optimization [19]. For the purposes of the current analysis, the acute treatment optimization grouping was merged to “poorly optimized” (“very poor” (score 0) and “poor” (score 1–5) groups), “optimized” (“moderate” (score 6–7) and “maximal” categories (score 8)), as this was previously applied elsewhere [20]; (ii) proportion of individual hypersensitivity symptoms accompanying headache [21], including osmophobia (dislike or aversion to smell or odors), photophobia (sensitivity or aversion to light), phonophobia (sensitivity or aversion to sounds), nausea/vomiting (urge to vomit/forceful ejection of the contents of the stomach through the mouth) and allodynia, i.e., pain generated after applying a non-noxious stimulus. For this cluster of the analysis, patients also provided numerical data concerning the changes in the average days with the specific symptoms between follow-ups; (iii) proportion of individual prodromal symptoms (premonitory symptoms that often precede a migraine attack), including mood changes, yawning, somnolence, drowsiness, food craving, neck stiffness and fatigue [22]; and (iv) presence of triggers followed by headache (endogenous or exogenous stimuli that lower the threshold for the onset of an attack in migraine-predisposed patients), including stress, irregular sleep schedule, specific food consumption, alcohol/caffeine intake, weather changes, dehydration and also luminous and olfactory stimuli [23]. Menstruation was not analyzed as a triggering factor of migraine in the current study.

We then compared the above-mentioned clinical variables from baseline to month 3, 6, and 9 between the merged non-responders and super non-responders (<50% reduction in MHDs) vs. merged responders and super responders (>50% MHD reduction) in order to seek the corresponding fremanezumab effects on triptan response, hypersensitivity and prodromal symptoms as well as on migraine triggers.

### Statistical Analysis

Descriptive statistics were generated for all clinical variables throughout the study period, depending on their nature. Patients responding to fremanezumab and non-responders were compared using the two-sided chi square test and Fisher’s exact test for categorical variables and with the Mann–Whitney test for numerical variables. Unless otherwise stated, all tests were two-sided, and the significance was set at the level of *p* < 0.05. SPSS for Windows (release 27.0; SPSS Inc., Chicago, IL, USA) was used to conduct the statistical analysis of the data, overall. 

## 3. Results

A total of 63 patients with HFEM were included in the current study. Their mean age was 46.1 ± 10.3 (22–60) years, while 49 (77.8%) of them were females. The mean time since migraine diagnosis was 20.1 ± 8.9 (2–40) years, and patients received a median number of four (three–seven) previous preventative medications. Their median baseline MHDs were at 12, while 17 (27%) patients had coexistent MOH at baseline, and 5 (7.9%) experienced aura. Among these 63 patients, 51 (81%) were classified as treatment responders (>50% reduction in MHDs), 24 obtaining responses at 50% and 27 at 75%, compared to baseline, whereas 12 remained unresponsive (8 for partial and 4 for super non-response). The demographic and clinical characteristics of the study’s population, according to their response to fremanezumab, are summarized in Table 1.

### 3.1. Longitudinal Effects of Fremanezumab on the Response to Triptans

All patients received triptan at baseline as abortive migraine therapy with various degrees of analgesic effects. However, after a 9-month continuous exposure to fremanezumab vs. baseline, responders at either 50% or 75% MHD reduction were significantly more likely to report adequate optimization of their acute migraine headache after triptans intake, compared with non-responders, according to the mTOQ-4 scorings. mTOQ-4 scoring increased from a median value of three to six in responders and from three to seven in super-responders at month 9, compared to baseline. Nonetheless, this beneficial effect was evident after the first 3 months of therapy (Table 2). Noteworthily, a significant percentage of non-responders (6/12; 50%) also reported some degree of response to triptans optimization while being on fremanezumab, compared to baseline (median mTOQ-4 scoring from two to six).

### 3.2. Longitudinal Effects of Fremanezumab on the Incidence of Self-Reported Hypersensitivity Symptoms Accompanying Headache

Typical migraine hypersensitivity symptoms accompanying headache, including osmophobia, photophobia, phonophobia, nausea/vomiting and allodynia occurred at baseline numerically more frequently in non-responders than in responders, without, though, reaching statistical significance. Excepting allodynia, fremanezumab significantly reduced the monthly average number of days of all other migraine-associated symptoms in responders vs. non-responders at month 9, compared to baseline. Osmophobia was reduced by a median of 4 days in responders vs. 2 days in non-responders (*p* = 0.03), nausea or vomiting by a median of 10 vs. 3 days in non-responders (*p* < 0.001), while similar significant reductions were seen for photophobia and phonophobia between responders and non-responders at month 9 compared to baseline (−8 vs. −3 and −9 vs. −3 days for photophobia and phonophobia, respectively). The median monthly days with allodynia remained comparable between the response groups (*p* = 0.232). These beneficial effects in the reduction in median days with migraine-associated symptoms were mostly seen after 6 months of therapy with fremanezumab.

In line with the latter significant reductions in monthly days with migraine-associated symptoms, their crude incidence also numerically decreased in responders vs. non-responders to reach significance only for osmophobia at month 9 compared to baseline (*p* = 0.016) and nausea/vomiting at month 6 (*p* = 0.033) and 9 (*p* = 0.03) compared to baseline (Table 3), thoroughly demonstrating that fremanezumab was able to reduce the incidence of migraine-associated symptoms mostly in treatment responders but also in those patients with an at least 30% MHD reduction. On the contrary, super non-responders remained with the same incidence of all migraine-associated hypersensitivity symptoms during the 9-month treatment period. 

### 3.3. Longitudinal Effects of Fremanezumab on the Incidence of Self-Reported Prodromal Symptoms Followed by Headache

A total of 40 (63.5%) patients reported at baseline the presence of premonitory symptoms followed by headache at various incidences and a median value of three (Table 4). Mood changes, yawning and fatigue were the most frequent patient-reported migraine prodromes. However, at the last clinical follow-up performed after 9 months of therapy, it was evident that all prodromal symptoms followed by headache decreased by 60% in the responders and literally remained unchanged in the non-responders. 

After treatment with fremanezumab, the occurrence of all prodromal symptoms was significantly reduced, in the responders compared to baseline, to being scarcely reported. Significant reductions were seen for mood changes (*p* = 0.004), yawning (*p* = 0.009), somnolence (*p* = 0.006), neck stiffness (*p* = 0.006) and fatigue (*p* = 0.004) between responders and non-responders at month 9 compared to baseline (Table 4). For some of these symptoms, i.e., yawning, somnolence and neck stiffness, significant reductions in their incidences between responders and non-responders were seen after 6 months of therapy, while at month 3, no such improvements were substantiated. Noteworthily, a significant percentage of partial non-responders also reported a marked reduction in the incidence of the majority of the analyzed pre-monitory symptoms, including mood changes (−50%), somnolence (−50%) and neck stiffness (−66.6%). Fremanezumab had no effects on the incidence of prodromal symptoms of super non-responders (Table 4).

### 3.4. Longitudinal Effects of Fremanezumab on Various Self-Reported Migraine Triggers

After treatment with fremanezumab, the overall incidence of triggers to evoke headache in the responders compared with non-responders dropped by 33% and 3%, respectively (*p* < 0.001). This reduction was mostly seen in the group of super-responders. However, there were no substantial improvements in the occurrence of triggers between responders and non-responders at month 3, 6 or 9, compared to baseline (Table 5). 

## 4. Discussion

No more than five studies, so far, have specifically elucidated the effects of anti-CGRP Mabs on the incidence of triggers and also on prodromal and migraine-associated symptoms. Whether the response to triptans would also change after prophylactic treatment with either fremanezumab or galcanezumab (both target the CGRP ligand) remains yet scarcely reported [24,25]. In order to likely provide additional relevant data to the latter partly clarified clinical issues, we longitudinally studied for a time period of a continuous 9-month treatment a homogenous sample of patients with HFEM to seek if fremanezumab would be able to significantly reduce the incidence of migraine symptoms other than the headache or the severity itself. We also aimed to ascertain if fremanezumab would exert neuromodulatory effects when administered for 9 monthly cycles. Our study was the first to provide such data longitudinally obtained at month 3, 6 and 9 (three time points), compared to baseline, opposed to the recently published study with galcanezumab use, wherein patients were followed at just a single and much shorter time point after 3 months of treatment versus pre-treatment [13]. 

Although early effects usually occur with fremanezumab use, late effects with anti-CGRP Mabs may occur in about 15% patients even after 12 months of treatment [15,26]. We herein demonstrated that fremanezumab had early (after 3 monthly cycles) beneficial effects on the response to triptans in the majority of responders but also in half of the partial non-responders. A significant reduction in median days with migraine-associated symptoms was seen in responders after 6 months of therapy with fremanezumab, mostly for osmophobia, photophobia, phonophobia and nausea/vomiting, but partial non-responders (30–49% MHD reduction) also benefited. Same patterns of response were observed for pre-monitory symptoms with significant reductions in their incidence in responders and modest patients, though with clinically relevant effects in partial non-responders, while the incidence of triggers remained essentially unaffected in both responders and non-responders.

Our results were in agreement with previous studies demonstrating that Mabs targeting the CGRP ligand, i.e., fremanezumab and galcanezumab, were able to prevent not only migraine headache but also its prodromal and accompanying symptoms [12,13,27,28]. However, concerning the trigger reduction followed by headache, we failed to confirm the results of another recently published study with galcanezumab use, which demonstrated a significant decrease in the incidence of migraine prodromal and associated symptoms but also reductions in the number of triggers between responders (38%) and non-responders (13%), as also between super-responders (31%) and super non-responders (4%) [13]. To our knowledge, there are no published data on the impact of fremanezumab on migraine triggers. Nonetheless, we confirmed previous findings, demonstrating that starting erenumab in episodic and chronic migraine patients could improve triptan responses in treatment responders [29].

In line with our results, prodromes can frequently occur in up to 86% of migraine patients, while fatigue, concentration difficulties and mood changes are the most commonly reported pre-monitory symptoms, based on the results of a recently published systematic review and meta-analysis [30]. The importance of prodromes that can be used to identify the onset of migraine has recently attracted significant attention, based on a rational hypothesis suggesting that an early intervention in the migraine attack may prevent its onset or lessen its severity [5]. The pathophysiological phenomena accounting for the generation of the prodromal migraine phase are quite complex, but mostly involve dopaminergic dysfunction [31]; overactivity in the hypothalamic, brainstem and diencephalic systems, as also in the occipital cortex [32] and abnormal connectivity of different brain regions, including the cortex, thalamus, hypothalamus, brainstem, amygdala and cerebellum [33]. Central sensitization involving the trigeminocervical complex may contribute to the neck stiffness/pain that is often reported among migraine prodromes [34]. As such, it is conceived that fremanezumab is able to reduce the hyperactivity in the corresponding brain and deeper cervical areas through CGRP inhibition to result in a clinically meaningful reduction in premonitory symptoms of our responders and partial non-responders. In support of our assumption are the results of a recently published study with another Mab targeting the CGRP ligand, which aimed to determine the effects of a 3-month treatment with galcanezumab on the cortical thickness of patients with HFEM or chronic migraine with the use of high-resolution magnetic resonance imaging. This study demonstrated that galcanezumab was able to alter cortical gray matter thickness (compared to baseline) in the responders, thoroughly evoking a reduction in the number of pain/nociceptive signals as a result of maladaptive neural activity to actually reflect a recovery process from neural swelling and dendritic complexity [35]. Although not yet specifically documented, it has been suggested that fremanezumab may exert similar morphological changes in the migraine brain as those seen with galcanezumab. 

The mechanisms by which migraine-associated symptoms are generated have not been fully elucidated. Nonetheless, it has been previously demonstrated that the overactivity of the occipital cortex and brainstem could be responsible for the manifestations of osmophobia, photophobia, phonophobia and nausea [36,37], while the central sensitization processes, coupled with changes in the connectivity of overlapping brain circuits, may contribute to the generation of allodynia and hypersensitivity accompanying headache [38,39]. Again, it has been suggested that fremanezumab-associated reduced neuronal hyperexcitability may account for the reduction in premonitory symptoms of responders and partial non-responders. 

Although, pathophysiological mechanisms involving abnormal neuronal excitability in the migraine brain may also be responsible for the triggers of a migraine attack through the activation of meningeal nociceptors by external stimuli [40], we were unable to demonstrate (as prementioned) that fremanezumab can impact the incidence of triggers, contrary to findings of beneficial effects on migraine triggers after galcanezumab exposure [13]. Obviously, methodological discrepancies may have accounted for the different results, including the inclusion of a mixed population of episodic and chronic migraine patients treated with galcanezumab for 3 months, compared to our study wherein a homogenous sample of HFEM patients was longitudinally followed-up with during 9 months of continuous fremanezumab prophylactic treatment. 

## 5. Conclusions

Considering some methodological limitations of our study, including the modest sample size allowing univariate but not multivariate comparisons, the open-label design and lack of data on postdromal symptoms, coupled with difficulties with reliable documentation of migraine-associated prodromal and hypersensitivity symptoms from patients’ self-reporting, we conclude that fremanezumab treatment continuously given for an adequate time period of at least 6–9 months may exert neuromodulatory effects in the migraine brain. These effects may result in both the inhibition of migraine escalation from HFEM to chronic migraine and also in the diminishing of the magnitude of migraine-associated prodromal and hypersensitivity symptoms, mostly in treatment responders at 50% and 75% but also in those with a 30% MHD reduction (partial non-responders). Alterations in CGRP signaling and the blockade of repetitive nociceptive signal transduction from the periphery to central brain structures, as well as circuits receiving trigeminovascular input, seem to hold responsibility for the beneficial clinical effects of fremanezumab, as we herein demonstrated. 

## Figures and Tables

**Table 1:** Baseline demographic and clinical characteristics of enrolled patients (*n* = 63), stratified according to their response to fremanezumab.

	Responders (50–74% MHD Reduction) *n* = 24	Non-Responders (30–49% MHD Reduction) *n* = 8	Super Responders (75–100% MHD Reduction) *n* = 27	Super Non-Responders (0–29% MHD Reduction) *n* = 4
**Age** in years ± SD (range)	47.7 ± 10.1 (22–60)	52.5 ± 6.6 (32–58)	41.2 ± 9.9 (25–58)	55.5 ± 3.1 (20–58)
**Gender** (females over males)	16 (66.7%)	8 (100%)	21 (77.8%)	4 (100%)
**Years with migraine** ± SD (range)	20.3 ± 10.3 (3–40)	27.2 ± 6.9 (12–40)	18.1 ± 6.1 (8–30)	21.2 ± 8.5 (10–35)
**Number of previous preventative medication lines** Median value (range)	4 (3–7)	3 (3–5)	4 (3–7)	4 (3–7)
**MHD** (median)	12	13	12	12.5
**MOH** (no over yes)	19 (79.2%)	5 (62.5%)	20 (74.1%)	2 (50%)
**Aura** (no over yes)	20 (83.3%)	8 (100%)	27 (100%)	3 (75%)

Abbreviations: MHDs: monthly headache days; MOH: medication-overuse headache.

**Table 2:** Longitudinal effects of fremanezumab on triptan response.

	Responders (50–74% MHD Reduction) *n* = 24	Non-Responders (30–49% MHD Reduction) *n* = 8	Super Responders (75–100% MHD Reduction) *n* = 27	Super Non-Responders (0–29% MHD Reduction) *n* = 4	*p* Value ^#^ between Responders and Non-Responders *
**Response to triptans**					
Baseline *n* (%)	17 (70.8)	0 (100)	10 (37.0)	0 (100)	
After 3 months	20 (83.3)	0 (100)	24 (88.9)	0 (100)	**<0.001**
After 6 months	24 (100)	2 (25.0)	25 (92.6)	0 (100)	**<0.001**
After 9 months	24 (100)	5 (62.5)	26 (96.3)	1 (25.0)	**<0.001**

Abbreviations: MHDs: monthly headache days; *p* value ^#^ calculated with the chi-square test and Fisher’s exact test. * Longitudinal comparison between groups of merged non-responders and super non-responders (<50% reduction in MHDs—*n* = 12) vs. merged responders and super responders (>50% MHD reduction—*n* = 51) at either month 3, 6 or 9, compared to baseline. *p* values in bold indicate statistical significance.

**Table 3:** Longitudinal effects of fremanezumab on hypersensitivity symptoms, according to treatment response.

	Responders (50–74% MHD Reduction) *n* = 24	Non-Responders (30–49% MHD Reduction) *n* = 8	Super Responders (75–100% MHD Reduction) *n* = 27	Super Non-Responders (0–29% MHD Reduction) *n* = 4	*p* Value ^#^ between Responders and Non-Responders *
**Osmophobia**					
Baseline *n* (%)	8 (33.3)	2 (25)	14 (51.9)	2 (50)	
After 3 months	8 (33.3)	2 (25)	14 (51.9)	2 (50)	0.752
After 6 months	7 (29.2)	2 (25)	6 (22.2)	2 (50)	0.159
After 9 months	4 (16.6)	2 (25)	4 (14.8)	2 (50)	**0.016**
**Phonophobia**					
Baseline *n* (%)	12 (50)	5 (62.5)	20 (74.1)	4 (100)	
After 3 months	12 (50)	5 (62.5)	17 (63)	4 (100)	>0.999
After 6 months	8 (33.3)	3 (37.5)	14 (51.9)	4 (100)	0.752
After 9 months	2 (8.3)	2 (25)	12 (44.4)	4 (100)	0.752
**Photophobia**					
Baseline *n* (%)	12 (50)	6 (75)	20 (74.1)	4 (100)	
After 3 months	8 (33.3)	5 (62.5)	14 (51.9)	4 (100)	0.752
After 6 months	8 (33.3)	3 (37.5)	13 (48.1)	4 (100)	0.752
After 9 months	2 (8.3)	2 (25)	12 (44.4)	4 (100)	0.172
**Nausea/vomiting**					
Baseline *n* (%)	14 (58.2)	5 (62.5)	18 (66.7)	4 (100)	
After 3 months	12 (50)	5 (62.5)	15 (55.6)	4 (100)	0.343
After 6 months	8 (33.3)	5 (62.5)	7 (25.9)	4 (100)	**0.033**
After 9 months	3 (12.5)	5 (62.5)	7 (25.9)	4 (100)	**0.030**
**Allodynia**					
Baseline *n* (%)	6 (25)	4 (50)	7 (25.9)	4 (100)	
After 3 months	6 (25)	4 (50)	6 (22.2)	4 (100)	0.751
After 6 months	5 (20.8)	4 (50)	5 (18.5)	4 (100)	0.652
After 9 months	5 (20.8)	4 (50)	4 (14.8)	4 (100)	0.630

Abbreviations: MHDs: monthly headache days; *p* value ^#^ calculated with the chi-square test and Fisher’s exact test. * Longitudinal comparison between groups of merged non-responders and super non-responders (<50% reduction in MHDs—*n* = 12) vs. merged responders and super responders (>50% MHD reduction—*n* = 51) at month 3, 6 or 9, compared to baseline. *p* values in bold indicate statistical significance.

**Table 4:** Longitudinal effects of fremanezumab on prodromal symptoms, according to treatment response.

	Responders (50–74% MHD Reduction) *n* = 24	Non-Responders (30–49% MHD Reduction) *n* = 8	Super Responders (75–100% MHD Reduction) *n* = 27	Super Non-Responders (0–29% MHD Reduction) *n* = 4	*p* Value ^#^ between Responders and Non-Responders *
**Mood changes**					
Baseline *n* (%)	8 (33.3)	4 (50)	17 (63)	4 (100)	
After 3 months	8 (33.3)	2 (25)	14 (51.9)	4 (100)	0.752
After 6 months	8 (33.3)	2 (25)	14 (51.9)	4 (100)	0.752
After 9 months	1 (4.2)	2 (25)	4 (14.8)	4 (100)	**0.004**
**Yawning**					
Baseline *n* (%)	8 (33.3)	4 (50)	16 (59.3)	4 (100)	
After 3 months	8 (33.3)	3 (37.5)	13 (48.1)	3 (75)	0.747
After 6 months	1 (4.2)	2 (25)	3 (11.1)	3 (75)	**0.009**
After 9 months	1 (4.2)	2 (25)	3 (11.1)	3 (75)	**0.009**
**Somnolence**					
Baseline *n* (%)	7 (29.2)	0 (0)	13 (48.1)	3 (75)	
After 3 months	1 (4.2)	0 (0)	8 (29.6)	3 (75)	0.249
After 6 months	0 (0)	0 (0)	8 (29.6)	3 (75)	**0.006**
After 9 months	0 (0)	0 (0)	8 (29.6)	3 (75)	**0.006**
**Drowsiness**					
Baseline *n* (%)	1 (4.2)	2 (25)	8 (29.6)	3 (75)	
After 3 months	1 (4.2)	2 (25)	8 (29.6)	3 (75)	>0.999
After 6 months	1 (4.2)	2 (25)	8 (29.6)	3 (75)	>0.999
After 9 months	1 (4.2)	2 (25)	8 (29.6)	3 (75)	>0.999
**Food craving**					
Baseline *n* (%)	2 (8.3)	3 (37.5)	14 51.9)	4 (100)	
After 3 months	2 (8.3)	3 (37.5)	11 (40.7)	4 (100)	0.684
After 6 months	1 (4.2)	3 (37.5)	10 (37.1)	4 (100)	0.684
After 9 months	1 (4.2)	3 (37.5)	10 (37.1)	3 (75)	0.680
**Neck stiffness**					
Baseline *n* (%)	6 (25)	3 (37.5)	2 (7.4)	3 (75)	
After 3 months	6 (25)	2 (25)	2 (7.4)	3 (75)	0.425
After 6 months	0 (0)	2 (25)	0 (0)	3 (75)	**0.006**
After 9 months	0 (0)	1 (12.5)	0 (0)	3 (75)	**0.006**
**Fatigue**					
Baseline *n* (%)	8 (33.3)	4 (50)	17 (63)	4 (100)	
After 3 months	8 (33.3)	2 (25)	14 (51.9)	4 (100)	0.752
After 6 months	8 (33.3)	3 (37.5)	13 (48.1)	3 (75)	0.747
After 9 months	1 (4.2)	2 (25)	4 (14.8)	4 (100)	**0.004**

Abbreviations: MHDs: monthly headache days; *p* value ^#^ calculated with the chi-square test and Fisher’s exact test. * Longitudinal comparison between groups of merged non-responders and super non-responders (<50% reduction in MHDs—*n* = 12) vs. merged responders and super responders (>50% MHD reduction—*n* = 51) at month 3, 6 or 9, compared to baseline. *p* values in bold indicate statistical significance.

**Table 5:** Longitudinal effects of fremanezumab on triggers followed by headache, according to treatment response.

	Responders (50–74% MHD Reduction) *n* = 24	Non-Responders (30–49% MHD Reduction) *n* = 8	Super Responders (75–100% MHD Reduction) *n* = 27	Super Non-Responders (0–29% MHD Reduction) *n* = 4	*p* Value ^#^ between Responders and Non-Responders *
**Stress**					
Baseline *n* (%)	20 (83.3)	8 (100)	18 (66.6)	4 (100)	
After 3 months	18 (75)	8 (100)	15 (15.5)	4 (100)	0.252
After 6 months	15 (62.5)	8 (100)	15 (15.5)	4 (100)	0.250
After 9 months	12 (50)	8 (100)	15 (15.5)	4 (100)	0.152
**Irregular sleep**					
Baseline *n* (%)	12 (50)	6 (75)	15 (55.5)	4 (100)	
After 3 months	12 (50)	6 (75)	15 (55.5)	4 (100)	0.751
After 6 months	6 (25)	6 (75)	15 (55.5)	4 (100)	0.356
After 9 months	6 (25)	6 (75)	15 (55.5)	4 (100)	0.356
**Specific food consumption**					
Baseline *n* (%)	8 (33.3)	2 (25)	12 (44.4)	3 (75)	
After 3 months	8 (33.3)	2 (25)	12 (44.4)	3 (75)	>0.999
After 6 months	8 (33.3)	2 (25)	12 (44.4)	3 (75)	>0.999
After 9 months	8 (33.3)	2 (25)	10 (37.1)	3 (75)	>0.999
**Alcohol/caffeine**					
Baseline *n* (%)	7 (29.2)	6 (75)	8 (29.6)	2 (50)	
After 3 months	7 (29.2)	6 (75)	8 (29.6)	2 (50)	>0.999
After 6 months	7 (29.2)	6 (75)	7 (25.9)	2 (50)	>0.999
After 9 months	6 (25)	5 (62.5)	7 (25.9)	2 (50)	>0.999
**Weather changes**					
Baseline *n* (%)	6 (25)	5 (62.5)	7 (25.9)	2 (50)	
After 3 months	6 (25)	5 (62.5)	6 (22.2)	2 (50)	>0.999
After 6 months	6 (25)	5 (62.5)	6 (22.2)	2 (50)	>0.999
After 9 months	6 (25)	5 (62.5)	6 (22.2)	2 (50)	>0.999
**Dehydration**					
Baseline *n* (%)	9 (37.5)	4 (50)	6 (22.2)	0 (0)	
After 3 months	9 (37.5)	4 (50)	6 (22.2)	0 (0)	>0.999
After 6 months	9 (37.5)	4 (50)	6 (22.2)	0 (0)	>0.999
After 9 months	8 (33.3)	4 (50)	6 (22.2)	0 (0)	>0.999
**Luminous/olfactory stimuli**					
Baseline *n* (%)	10 (41.7)	4 (50)	8 (29.6)	4 (100)	
After 3 months	10 (41.7)	4 (50)	8 (29.6)	4 (100)	0.561
After 6 months	6 (25)	4 (50)	6 (22.2)	4 (100)	0.231
After 9 months	6 (25)	4 (50)	6 (22.2)	4 (100)	0.231

Abbreviations: MHD: monthly headache days; *p* value ^#^ calculated with the chi-square test and Fisher’s exact test. * Longitudinal comparison between groups of merged non-responders and super non-responders (<50% reduction in MHDs—*n* = 12) vs. merged responders and super responders (>50% MHD reduction—*n* = 51) at months 3, 6 or 9, compared to baseline.

## Data Availability

The data that support the findings of this study are available from the corresponding author upon reasonable request.

## References

[1] (2018). Headache Classification Committee of the International Headache Society (IHS) The International Classification of Headache Disorders, 3rd edition. Cephalalgia.

[2] Argyriou A.A., Mantovani E., Mitsikostas D.-D., Vikelis M., Tamburin S. (2022). A systematic review with expert opinion on the role of gepants for the preventive and abortive treatment of migraine. Expert. Rev. Neurother..

[3] Eigenbrodt A.K., Christensen R.H., Ashina H., Iljazi A., Christensen C.E., Steiner T.J., Lipton R.B., Ashina M. (2022). Premonitory symptoms in migraine: A systematic review and meta-analysis of observational studies reporting prevalence or relative frequency. J. Headache Pain.

[4] Mollaoğlu M. (2013). Trigger factors in migraine patients. J. Health Psychol..

[5] Quintela E., Castillo J., Muñoz P., Pascual J. (2006). Premonitory and resolution symptoms in migraine: A prospective study in 100 unselected patients. Cephalalgia.

[6] Charbit A.R., Akerman S., Goadsby P.J. (2010). Dopamine: What′s new in migraine?. Curr. Opin. Neurol..

[7] Asghar M., Hansen A., Kapijimpanga T., van der Geest R., van der Koning P., Larsson H., Olesen J., Ashina M. (2010). Dilation by CGRP of middle meningeal artery and reversal by sumatriptan in normal volunteers. Neurology.

[8] Edvinsson L., Haanes K.A., Warfvinge K., Krause D.N. (2018). CGRP as the target of new migraine therapies—Successful translation from bench to clinic. Nat. Rev. Neurol..

[9] Edvinsson L. (2017). The Trigeminovascular Pathway: Role of CGRP and CGRP Receptors in Migraine. Headache.

[10] Tepper S.J. (2018). History and review of anti-calcitonin gene-related peptide (CGRP) therapies: From translational research to treatment. Headache.

[11] Basedau H., Sturm L.M., Mehnert J., Peng K.P., Schellong M., May A. (2022). Migraine monoclonal antibodies against CGRP change brain activity depending on ligand or receptor target—An fMRI study. eLife.

[12] Iannone L.F., De Cesaris F., Ferrari A., Benemei S., Fattori D., Chiarugi A. (2022). Effectiveness of anti-CGRP monoclonal antibodies on central symptoms of migraine. Cephalalgia.

[13] Ashina S., Melo-Carrillo A., Toluwanimi A., Bolo N., Szabo E., Borsook D., Burstein R. (2023). Galcanezumab effects on incidence of headache after occurrence of triggers, premonitory symptoms, and aura in responders, non-responders, super-responders, and super non-responders. J. Headache Pain.

[14] Argyriou A.A., Dermitzakis E.V., Xiromerisiou G., Rallis D., Soldatos P., Litsardopoulos P., Vikelis M. (2023). Efficacy and safety of fremanezumab for migraine prophylaxis in patients with at least three previous preventive failures: Prospective, multicenter, real-world data from a Greek registry. Eur. J. Neurol..

[15] Barbanti P., Egeo G., Aurilia C., Torelli P., Finocchi C., D’onofrio F., D’onofrio L., Rao R., Messina S., Di Clemente L. (2023). Early and sustained efficacy of fremanezumab over 24-weeks in migraine patients with multiple preventive treatment failures: The multicenter, prospective, real-life FRIEND2 study. J. Headache Pain.

[16] Argyriou A.A., Dermitzakis E.V., Xiromerisiou G., Rallis D., Soldatos P., Litsardopoulos P., Vikelis M. (2023). Predictors of Response to Fremanezumab in Migraine Patients with at Least Three Previous Preventive Failures: Post Hoc Analysis of a Prospective, Multicenter, Real-World Greek Registry. J. Clin. Med..

[17] Vikelis M., Dermitzakis E.V., Xiromerisiou G., Rallis D., Soldatos P., Litsardopoulos P., Rikos D., Argyriou A.A. (2023). Effects of Fremanezumab on Psychiatric Comorbidities in Difficult-to-Treat Patients with Chronic Migraine: Post Hoc Analysis of a Prospective, Multicenter, Real-World Greek Registry. J. Clin. Med..

[18] Sacco S., Bendtsen L., Ashina M., Reuter U., Terwindt G., Mitsikostas D.D., Martelletti P. (2019). European headache federation guideline on the use of monoclonal antibodies acting on the calcitonin gene related peptide or its receptor for migraine prevention. J. Headache Pain.

[19] Lipton R.B., Kolodner K., Bigal M.E., Valade D., Láinez M.J., Pascual J., Gendolla A., Bussone G., Islam N., Albert K. (2009). Validity and reliability of the Migraine-Treatment Optimization Questionnaire. Cephalalgia.

[20] Cady R., Lipton R.B., Buse D.C., Josiassen M.K., Lindsten A., Ettrup A. (2022). Optimization of acute medication use following eptinezumab initiation during a migraine attack: Post hoc analysis of the RELIEF study. J. Headache Pain.

[21] Villar-Martinez M.D., Goadsby P.J. (2022). Pathophysiology and Therapy of Associated Features of Migraine. Cells.

[22] Barbanti P., Fofi L., Aurilia C., Egeo G. (2013). Dopaminergic symptoms in migraine. Neurol. Sci..

[23] Kelman L. (2007). The triggers or precipitants of the acute migraine attack. Cephalalgia.

[24] Schoenen J., Van Dycke A., Versijpt J., Paemeleire K. (2023). Ten open questions in migraine prophylaxis with monoclonal antibodies blocking the calcitonin-gene related peptide pathway: A narrative review. J. Headache Pain.

[25] Vernieri F., Brunelli N., Marcosano M., Aurilia C., Egeo G., Lovati C., Favoni V., Perrotta A., Maestrini I., Rao R. (2023). Maintenance of response and predictive factors of 1-year GalcanezumAb treatment in real-life migraine patients in Italy: The multicenter prospective cohort GARLIT study. Eur. J. Neurol..

[26] Barbanti P., Aurilia C., Egeo G., Torelli P., Proietti S., Cevoli S., Bonassi S. (2023). Late Response to Anti-CGRP Monoclonal Antibodies in Migraine: A Multicenter Prospective Observational Study. Neurology.

[27] Brandes J.L., Kudrow D., Yeung P.P., Sakai F., Aycardi E., Blankenbiller T., Grozinski-Wolff M., Yang R., Ma Y. (2020). Effects of fremanezumab on the use of acute headache medication and associated symptoms of migraine in patients with episodic migraine. Cephalalgia.

[28] McAllister P., Lamerato L., Krasenbaum L.J., Cohen J.M., Tangirala K., Thompson S., Driessen M., Casciano J., Dotiwala Z., Mauskop A. (2021). Real-world impact of fremanezumab on migraine symptoms and resource utilization in the United States. J. Headache Pain.

[29] Frattale I., Caponnetto V., Casalena A., Assetta M., Maddestra M., Marzoli F., Affaitati G., Giamberardino M.A., Viola S., Gabriele A. (2021). Association between response to triptans and response to erenumab: Real-life data. J. Headache Pain.

[30] Christensen R.H., Eigenbrodt A.K., Ashina H., Steiner T.J., Ashina M. (2023). What proportion of people with migraine report postdromal symptoms? A systematic review and meta-analysis of observational studies. Cephalalgia.

[31] Barbanti P., Aurilia C., Egeo G., Fofi L., Guadagni F., Ferroni P. (2020). Dopaminergic symptoms in migraine: A cross-sectional study on 1148 consecutive headache center-based patients. Cephalalgia.

[32] Maniyar F.H., Sprenger T., Monteith T., Schankin C., Goadsby P.J. (2014). Brain activations in the premonitory phase of nitroglycerin-triggered migraine attacks. Brain.

[33] Amin F.M., Hougaard A., Magon S., Asghar M.S., Ahmad N.N., Rostrup E., Sprenger T., Ashina M. (2016). Change in brain network connectivity during PACAP38-induced migraine attacks: A resting-state functional MRI study. Neurology.

[34] Johnston M.M., Jordan S.E., Charles A.C. (2013). Pain referral patterns of the C1 to C3 nerves: Implications for headache disorders. Ann. Neurol..

[35] Szabo E., Ashina S., Melo-Carrillo A., Bolo N.R., Borsook D., Burstein R. (2023). Peripherally acting anti-CGRP monoclonal antibodies alter cortical gray matter thickness in migraine patients: A prospective cohort study. NeuroImage Clin..

[36] Maniyar F.H., Sprenger T., Schankin C., Goadsby P.J. (2014). Photic hypersensitivity in the premonitory phase of migraine--a positron emission tomography study. Eur. J. Neurol..

[37] Maniyar F.H., Sprenger T., Schankin C., Goadsby P.J. (2014). The origin of nausea in migraine-a PET study. J. Headache Pain.

[38] Schulte L.H., May A. (2016). The migraine generator revisited: Continuous scanning of the migraine cycle over 30 days and three spontaneous attacks. Brain.

[39] Chen Z., Chen X., Liu M., Dong Z., Ma L., Yu S. (2017). Altered functional connectivity of amygdala underlying the neuromechanism of migraine pathogenesis. J. Headache Pain.

[40] Marmura M.J. (2018). Triggers, Protectors, and Predictors in Episodic Migraine. Curr. Pain Headache Rep..

